# Effect of chronic alcohol and tobacco use on retinal nerve fibre layer thickness: a case–control study

**DOI:** 10.1136/bmjophth-2016-000003

**Published:** 2016-11-21

**Authors:** Shashi Ahuja, Praveen S Kumar, Vivek Praveen Kumar, Shivanand Kattimani, Sujiv Akkilagunta

**Affiliations:** 1 Department of Ophthalmology, Jawaharlal Institute of Postgraduate Medical Education and Research, Puducherry, India; 2 Department of Psychiatry, Jawaharlal Institute of Postgraduate Medical Education and Research, Puducherry, India; 3 Department of Preventive and Social Medicine, Jawaharlal Institute of Postgraduate Medical Education and Research, Puducherry, India

**Keywords:** Alcohol Use Disorders Identification Test (AUDIT), Fagerstorm Nicotine Dependence (FTND) scale, Optical coherence tomography, Tobacco-alcohol amblyopia

## Abstract

**Objective:**

To identify the effects of chronic alcohol and/or tobacco use on retinal nerve fibre layer (RNFL) thickness and to find the association between severity of addiction with RNFL thinning.

**Methodology:**

A case–control study was performed in 200 eyes of cases and 200 healthy control eyes. Cases were recruited from deaddiction clinic having history of alcohol and/or tobacco use for at least 5 years. Severity of alcohol and tobacco was graded by Alcohol Use Disorders Identification Test (AUDIT) and Fagerstorm Nicotine Dependence (FTND) scale, respectively. Age-matched and gender-matched individuals attending ophthalmology outpatient department without addiction were recruited as controls. RNFL thickness was measured using Stratus optical coherence tomography (OCT).

**Results:**

Statistically significant RNFL thinning was noted in all quadrants except nasal quadrant in the cases. Statistically significant thinning was seen in all quadrants except nasal with increased FTND scale. Thinning was noted in all quadrants with higher AUDIT scale, but this was statistically not significant.

**Conclusion:**

Chronic alcohol and tobacco use are likely to cause RNFL thinning. OCT can be used as a screening tool to suspect visual morbidities in chronic tobacco and alcohol users.

**Key messages**

- Alcohol and tobacco usages cause toxic optic neuropathy, that is, toxin-induced optic nerve insult. It primarily leads to axonal loss causing thinning of retinal nerve fibre layer (RNFL).
- We report that it is not only the presence of addiction of tobacco and alcohol but also the severity that causes thinning of RNFL, which can be detected preclinically by optical coherence tomography (OCT).
- Hence, all chronic alcoholics and tobacco users should undergo complete ophthalmic evaluation and assessment of RNFL by OCT.

## Introduction

Psychoactive substance abuse in India continues to be a substantive problem for the individual as well as for the society. Of the various substances, alcohol and tobacco are most commonly abused substances with majority of the people being dependent on them. The current prevalence of alcohol usage in India ranges from 65.84% to 67.4%.^1 2^ It varies in urban and rural areas with estimates being 7.3 per 1000 in urban areas to 5.8 per 1000 in rural areas.^2^ It is also common among the poorer sections of the society. Alcohol use in India is reported exclusively in males. Owing to its large population, India is the third largest market for alcoholic beverages in the world.^1^

Overall, tobacco use currently causes about six million deaths worldwide.^3^ Nearly one million deaths in a year in our country are attributable to smoking.^4^ According to the National Family Health Survey-3, tobacco usage is more prevalent among men, rural population, illiterates and poorer sections of the society.^5^ Tobacco usage inflicts high direct and indirect costs on the society due to mortality and morbidity resulting from their consumption. According to Indian Council of Medical Research, the total losses in 1999 due to tobacco-related diseases were almost 277.6 billion rupees. Morbidity due to these substance usage produce indirect costs on the society that includes the cost of caregivers and value of work loss due to illness.^6^

International Agency for Research on Cancer monograph shows that chronic tobacco smoking causes cancers like that of oral cavity, nasopharynx, oropharynx and hypopharynx, nasal cavity and paranasal sinuses, larynx, oesophagus, stomach, pancreas, liver, kidney, ureter, urinary bladder, uterine cervix and bone marrow (myeloid leukaemia).^7^ Tobacco use also causes toxic amblyopia. Abundant scientific evidences exist to establish that exposure to tobacco smoke can cause disease, disability and death.^3^ Tobacco smoking is a powerful risk factor conducive to various eye diseases, including the development of cataract, glaucoma, retinal vascular disorders and age-related macular degeneration. Tobacco amblyopia is the most common ocular disease related to its use.^8^

Review of literature revealed a small case series establishing retinal nerve fibre layer (RNFL) thinning in chronic alcohol and tobacco users.^9^ Hence, a large case–control study was undertaken to report the changes in RNFL thickness in patients of chronic alcohol and tobacco use. Since the patients were recruited from the deaddiction clinic from a tertiary care hospital, the RNFL thickness may vary in patients with acute intoxication.

Toxic optic neuropathy refers to a group of medical disorders defined by visual impairment due to optic nerve damage resulting from exposure to a toxin. The exact pathogenesis of toxic optic neuropathy resulting from tobacco and alcohol is not established and is probably multifactorial, but cyanide in tobacco smoke and malnutrition due to alcohol intake appear to be the most important causative factors.^10^ Toxic optic neuropathy is usually an underdiagnosed disease entity and a large proportion of patients present at a stage when recovery of vision is not possible. The mechanism is the primary insult to mitochondria that disrupts the process of oxidative phosphorylation causing axonal loss, which preferentially affects the parvocellular neurons in papillomacular bundle, thus resulting in thinning of RNFL.^10 11^

Optical coherence tomography (OCT) is a non-contact imaging technology that  provides live histopathological images of the retinal layers. An early diagnosis of decreased RNFL thickness goes a long way in preventing blindness due to toxic optic neuropathy and can also guide the treating physician about the severity of the condition.

## Aims and objectives

This study was conducted to assess the thickness of RNFL in chronic alcohol and tobacco users and to correlate the degree of RNFL thinning with the severity of alcohol and tobacco use.

## Method

A case–control study was conducted from August 2012 to July 2014 in a tertiary care teaching postgraduate medical institute in southern India. Ethical clearance from the institutional review board was obtained.

The cases comprised patients attending the deaddiction clinic in department of psychiatry, with at least 5 years of alcohol and/or tobacco use. Severity of alcohol and tobacco use were graded by the treating psychiatrist by specific questionnaires called Alcohol Use Disorders Identification Test (AUDIT) scoring system for alcohol use and Fagerstorm Nicotine Dependence (FTND) scale for tobacco use.

Age-matched and gender-matched individuals without any addictions attending outpatient department in ophthalmology were taken as controls.

Patients who were on drugs like ethambutol and isoniazid; those with systemic illnesses like diabetes mellitus, renal failure, multiple sclerosis; those on disulfiram for chronic alcoholism and those with glaucomatous disc changes and other causes of disc pallor were excluded from the study. An informed consent was taken from all the study participants.

**Figure 1:**
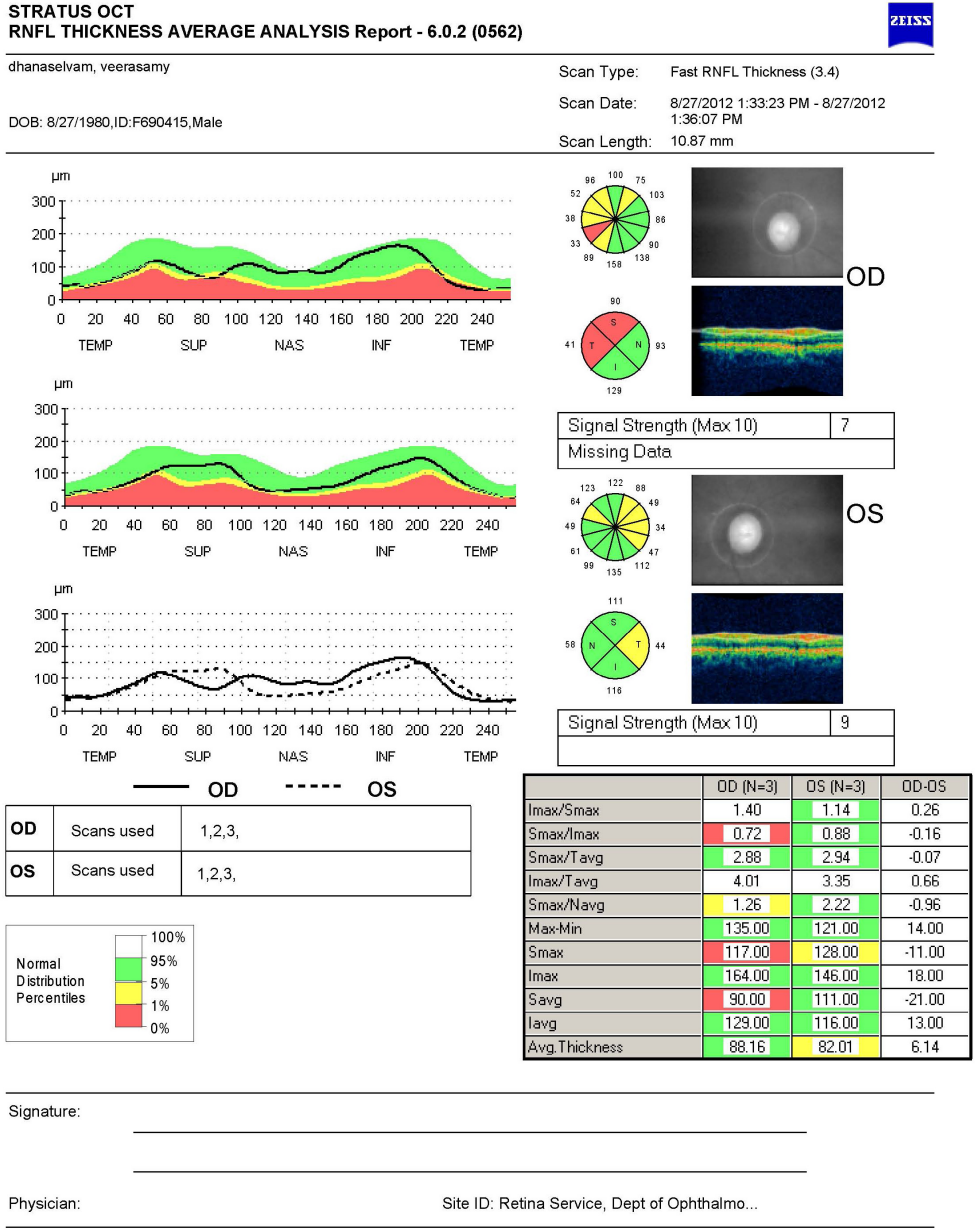
Fast retinal nerve fibre layer (RNFL) protocol optical coherence tomography (OCT) showing average RNFL thickness with false colour coding to depict the data

Demographic data such as age and gender were collected. Ocular complaint like diminution of vision was recorded. Ophthalmic examination included recording visual acuity by Snellen's chart and colour vision using the Ishiharas pseudoisochromatic plates. Detailed slit lamp examination was done. Pupillary reactions were noted. Fundus photograph was taken especially to look for temporal pallor of the optic disc. RNFL thickness was measured using time domain OCT (Stratus OCT; Carl Zeiss Meditec, Dublin, California, USA). Fast RNFL protocol was used to measure the peripapillary thickness (figure 1). Signal strength of six or more was taken as an acceptable scan. At least three scans were taken for each eye. The RNFL thickness parameters calculated by the Stratus OCT software (version 4.0.1) were averaged for thickness in the superior, inferior, temporal, and nasal quadrants. All cases and controls were worked up in the same way.

## Statistical analysis

Data analyses were done using STATA 11 (StataCorp, College Station, Texas, USA) and SPSS version 20. Participant characteristics were noted as proportion for categorical variables (visual acuity, FTND scale ≤5 and >5, AUDIT score ≤20 and >20) and mean±SD for continuous variables (RNFL thickness). Differences in RNFL thickness in each quadrant of the eyes among cases and controls were analysed using Mann- Whitney test. Statistical significance was considered at p≤0.05.

## Results

A total of 200 eyes of 100 cases and 200 eyes of 100 controls were included in the study. All the cases and controls were males with mean age of 39.5±10.1 years and 43.1±10.0 years, respectively.

Among 100 cases in the study, 9 (9%) patients were purely tobacco users in form of smoking and or tobacco chewing, 29 (29%) patients were purely alcohol users and 62 (62%) patients were both alcohol and tobacco users.

The best-corrected Snellen's visual acuity in all controls was 6/6, whereas in cases, the best-corrected visual acuity was 6/6 in 188 (94%) eyes and 12 (6%) eyes had decreased visual acuity. The visual acuity in these 12 eyes ranged from perception of light with accurate projection of rays to 6/18. Three eyes (25%) had visual acuity between 6/18 and 6/24, four eyes (33.33%) had visual acuity between 6/36 and 6/60 and five eyes (41.66%) had visual acuity of less than 6/60.

All patients in control group had a normal fundus findings and normal colour vision. Out of 200 eyes of cases, 12 eyes (6%) had optic disc pallor, which was more marked temporally. Out of these 12 eyes with disc pallor, red green colour deficiency were noted in 6 eyes, while remaining were having normal colour vision.

**Figure 2:**
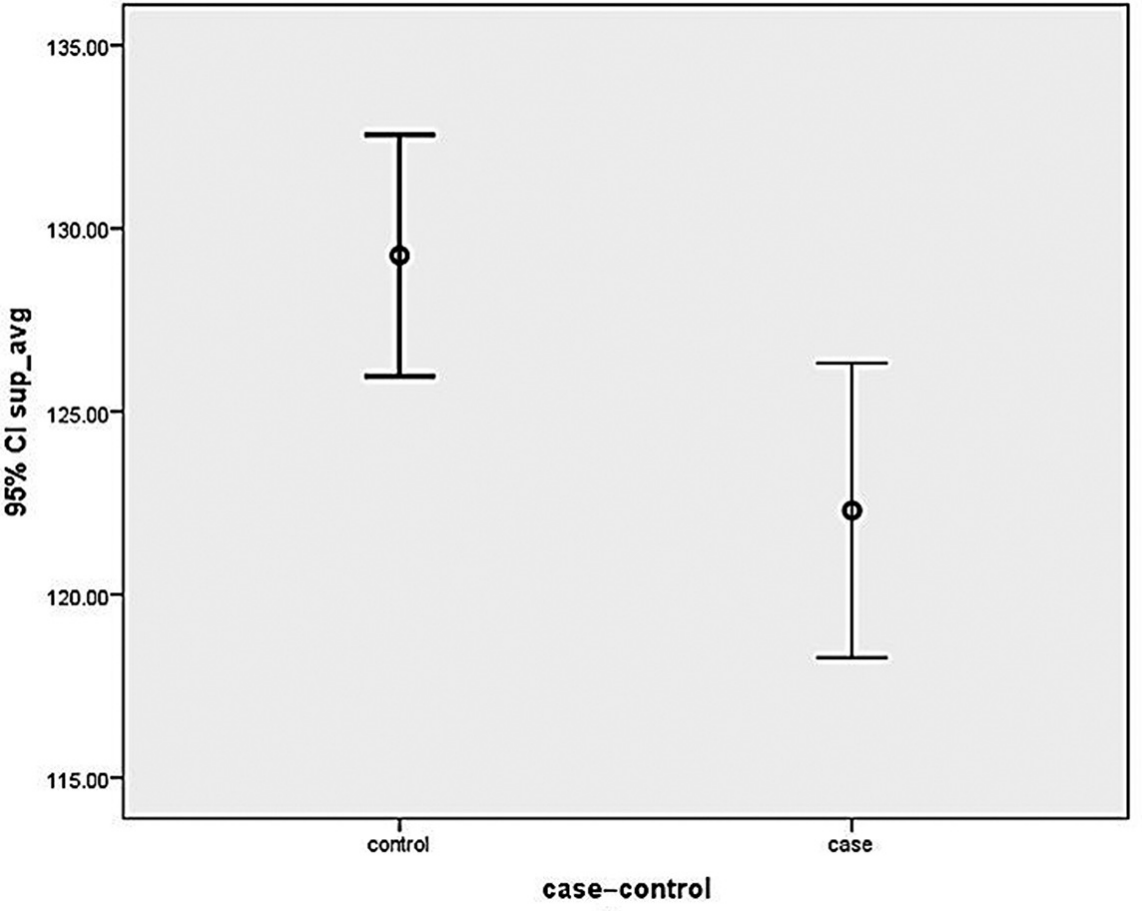
Graph showing mean retinal nerve fibre layer thickness in cases and controls in superior quadrant. CI, confidence interval.

**Figure 3:**
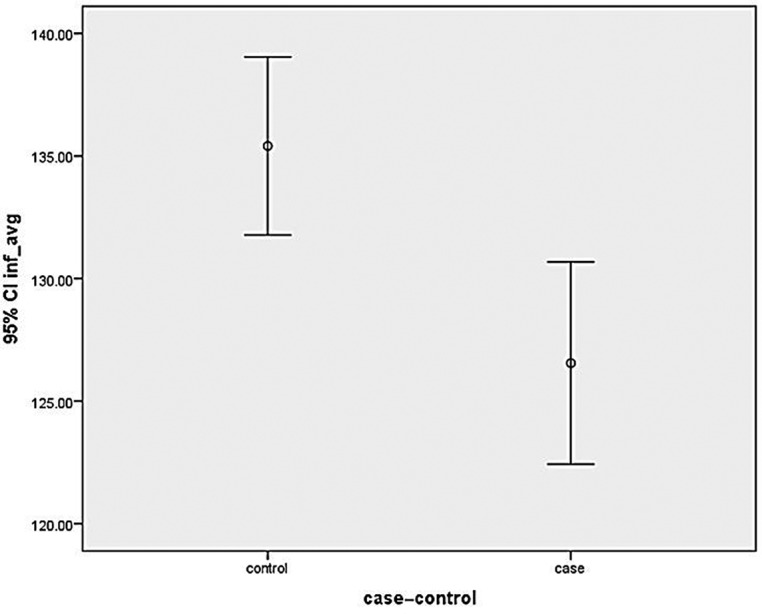
Graph showing mean retinal nerve fibre layer thickness in cases and controls in inferior quadrant. CI, confidence interval.

**Figure 4:**
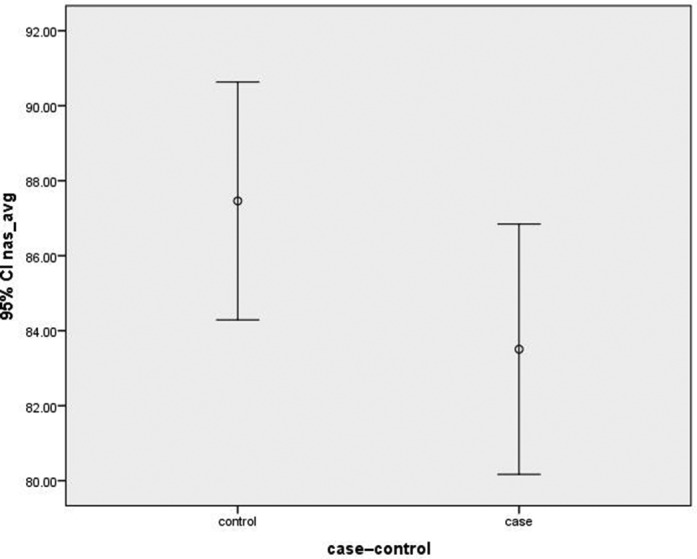
Graph showing mean retinal nerve fibre layer thickness in cases and controls in nasal quadrant. CI, confidence interval.

**Figure 5:**
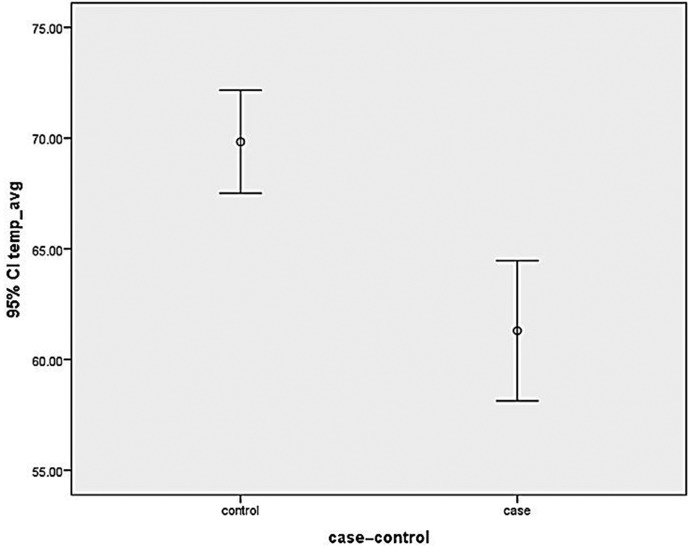
Graph showing mean retinal nerve fibre layer thickness in cases and controls in temporal quadrant. CI, confidence interval.

RNFL thickness was measured in all quadrants (ie, superior, inferior, nasal and temporal quadrants) of both eyes in cases and controls. Thinning of RNFL was noted in all quadrants (figures 2–5) of cases that  were statistically significant in superior, inferior and temporal quadrants when compared with controls (table 1).

**Table 1:** Retinal nerve fibre layer thickness (in microns) in all quadrants of eyes of cases and controls and their difference

Quadrant	Cases	Controls	p Value
Superior	122.30±20.26	129.2±16.69	0.005
Inferior	126.55±20.77	135.41±18.40	0.002
Nasal	83.50±16.81	87.46±16.06	0.081
Temporal	61.30±15.95	69.84±11.76	0.001

On comparing the RNFL thickness with the severity of alcohol use, greater thinning was noted in all quadrants in cases having AUDIT score more than 20 as compared with cases having AUDIT score ≤20, although they were statistically not significant (table 2).

**Table 2:** Retinal nerve fibre layer (RNFL) thickness in relation with Alcohol Use Disorders Identification Test (AUDIT) score in cases

Quadrant	RNFL thickness (in microns)		p Value
	AUDIT≤20	AUDIT>20	
Superior	121.61±16.28	122.51±21.31	0.620
Inferior	129.28±18.29	125.74±21.50	0.449
Nasal	81.72±14.42	81.04±17.52	0.342
Temporal	62.76±12.99	60.76±16.79	0.589

On comparing the RNFL thickness with severity of tobacco use, more thinning were noted in all quadrants in cases having FTND score >5 as compared with cases having FTND score ≤5, which was statistically significant in superior, inferior and temporal quadrants table 3.

**Table 3:** Retinal nerve fibre layer thickness (in microns) in all quadrants of eyes of cases and controls and their difference

Quadrant	RNFL thickness (in microns)		p Value
	FTND≤5	FTND>5	
Superior	126.80±18.25	111.29±21.18	0.007
Inferior	129.99±18.77	118.90±23.18	0.026
Nasal	85.30±16.11	79.50±17.90	0.215
Temporal	63.86±14.53	55.63±17.69	0.021

## Discussion

In this study, cases were younger than controls. This can be explained by the fact that people are likely to develop addictions at younger age group. This is in accordance with the study conducted by Girish *et al*,^2^ which shows that substance abuse is more common in young age groups.

All the cases were males. Singh *et al* ^12^ in their study on prevalence of regular alcohol users reported that 87.5% of males consumed alcohol daily.

This study showed that the visual acuity was decreased in only 12 eyes (6%) of cases. Remaining cases had normal visual acuity. This can be explained by the fact that tobacco alcohol amblyopia presents as slow painless, progressive, bilateral symmetric visual impairment. Since the patients were recruited from deaddiction clinic who were on abstinence, we found most of the patients with normal vision, which might progress to visual impairment with continued use of tobacco and alcohol. Hence, majority of the patients did not have diminution of vision.^11^

Out of 200 eyes of cases in our study, only 12 eyes (6%) had marked temporal disc pallor. Sharma *et al* ^11^ describe optic disc pallor to occur in later stages of the disease. Hence, in this study, most of the patients had normal fundus.

In this study, RNFL thickness was significantly less in all quadrants of cases compared with controls. This is in accordance with the study by Moura and  Monteiro^13^ who evaluated RNFL measurements in three cases of tobacco-alcohol-induced optic neuropathy using Stratus OCT and found thinning of RNFL in two cases. This is also in accordance with the study conducted by de Lima Rde *et al* ^9^ who evaluated the RNFL using GDx in chronic alcohol and tobacco users. The authors concluded that chronic use of tobacco and alcohol is associated with alteration of nerve fibre layer.

This study showed that there was RNFL thinning with increase in severity of tobacco use. This is because the mitochondrial insult in the disease process preferentially affects the fast-firing neurons in the papillomacular bundle resulting in thinning especially in temporal quadrant.

In this study, greater thinning of RNFL was observed in cases with more severe alcohol use although it was statistically not significant. This shows that tobacco affects the RNFL more as compared with alcohol.

Clinically more thinning of RNFL was noted in patients having diminution of vision compared with those patients with no ocular complaints, although statistical analysis could not be done as the number of patients having diminution of vision were less as compared with those with no ocular complaints.

None of the patients in our study showed increase in RNFL thickness. This can be explained by the fact that all the patients in the study were chronic alcohol and tobacco users and no patient reported to us with acute intoxication.

Strength of the study:

1. Large sample size: This study included large sample size of 100 cases and 100 controls, whereas previous studies had smaller sample size.
2. This study also correlated the severity of RNFL thinning with the severity of addiction. Other studies did not look for any correlation.
3. Our study also compared the visual morbidity with the RFNL thinning, which was not done earlier.

Limitations of the study:

Less number of pure alcoholics and pure tobacco users had been recruited in the study. This was primarily because synchronous users are more common. Hence, additive effect of each of these could not be addressed properly.

## Conclusions

Chronic use of tobacco and alcohol was found to cause thinning of RNFL in the eye. Increased severity of alcohol and tobacco use, that is, with higher FTND and AUDIT score is associated with greater thinning of RNFL. The authors recommend OCT as a screening tool to predict visual morbidity in patients with chronic tobacco and alcohol use.
